# Nucleoplasmic Nup98 controls gene expression by regulating a DExH/D-box protein

**DOI:** 10.1080/19491034.2017.1364826

**Published:** 2017-09-21

**Authors:** Juliana S. Capitanio, Ben Montpetit, Richard W. Wozniak

**Affiliations:** a Department of Cell Biology, University of Alberta, Edmonton, Canada; b Department of Viticulture and Enology, University of California at Davis, Davis, CA, USA

**Keywords:** DDX3, DDX5, DDX21, DExD/H-box helicases, DHX9, FG-Nup, gene expression regulation, nuclear pore complex, nucleoporins, Nup98

## Abstract

The nucleoporin Nup98 has been linked to the regulation of transcription and RNA metabolism,^1-3^ but the mechanisms by which Nup98 contributes to these processes remains largely undefined. Recently, we uncovered interactions between Nup98 and several DExH/D-box proteins (DBPs), a protein family well-known for modulating gene expression and RNA metabolism.^4-6^ Analysis of Nup98 and one of these DBPs, DHX9, showed that they directly interact, their association is facilitated by RNA, and Nup98 binding stimulates DHX9 ATPase activity.^7^ Furthermore, these proteins were dependent on one another for their proper association with a subset of gene loci to control transcription and modulate mRNA splicing.^7^ On the basis of these observations, we proposed that Nup98 functions to regulate DHX9 activity within the nucleoplasm.^7^ Since Nup98 is associated with several DBPs, regulation of DHX9 by Nup98 may represent a paradigm for understanding how Nup98, and possibly other FG-Nup proteins, could direct the diverse cellular activities of multiple DBPs.

## Dynamic nucleoporins and gene expression

Nucleoporins (Nups) are the protein building blocks of nuclear pore complexes (NPCs), structures that control the movement of cargo (e.g. proteins and RNA) across the nuclear envelope. In the last decade or so, Nups have also been found to influence genome organization and regulate gene expression, both within the context of NPCs and as distinct entities in the nucleoplasm.^2,8,9^ These dynamic Nup populations often move between the nucleoplasm, NPCs, and the cytoplasm.^10-12^ The mobility of several nucleoplasmic Nups, including Nup153, Nup98, Nup50 and sPom121, are known to be dependent on active RNA Polymerase II (RNA Pol II) transcription.^11-14^ These observations led to the suggestion that intranuclear Nup pools might actively participate in transcriptional regulation.^11,13,14^ A body of supporting evidence has further described interactions between Nups and transcriptional regulators, chromatin modifiers, and specific gene loci and transcripts (reviewed in^2,8,15^). In some instances, Nups interact with actively transcribed genes and/or groups of genes specific to development pathways and immune responses.^13,16-21^ These data, and others not discussed here due to space limitations, provide clear evidence that the cellular functions of many Nups extend beyond the confines of NPCs.

Among the intranuclear Nups, Nup98 has been widely studied, in part due to the observation of structural rearrangements of the *NUP98* gene in a variety of haematopoietic malignancies that lead to gene fusions containing the N-terminus of Nup98 (reviewed in^22^). The 920 amino-acid residue Nup98 protein contains multiple phenylalanine-glycine (FG) and glycine-leucine-phenylalanine-glycine (GLFG) repeats within the N-terminal half of the protein.^23^ This region is bisected by a small α-helical region that binds Rae1, an mRNA binding protein linked to mRNA export, and together the Rae1-Nup98 complex is capable of binding single-stranded RNA.^24^ The C-terminal half of Nup98 is thought to contain unstructured regions and a domain that mediates its association with other Nups within the NPC and in the nucleoplasm.^25-27^

At NPCs, Nup98 FG-repeats contribute multiple docking sites for nuclear transport factors as they move cargo through NPCs. Inside the nucleus, Nup98 is found distributed throughout the nucleoplasm and within intranuclear foci termed GLFG bodies.^11^ In multiple contexts, this intranuclear pool of Nup98 has been linked to the regulation of gene expression. For example, Drosophila Nup98 has been shown to interact with actively transcribing genes, with alterations in *NUP98* expression causing changes in the expression of hundreds of genes.^16,28^ Among these genes are tissue-specific *Hox* genes required for fly development.^16,18,28^ Nup98 and other intranuclear Nups also regulate the expression of developmental genes in mammalian cells, including genes functioning in cell differentiation and cell identity maintenance.^8,29^ Interestingly, Nup98 binds distinct genomic regions and influences the expression of diverse target genes in different human cell types and in pluri-/multi-potent cells, likely contributing to specific transcriptional programs related to cell identity.^14,30^ A key issue to address is how Nup98 is able to target these different gene subsets, which we postulate may involve the incorporation of Nup98 into effector complexes with different DBPs.

Nup98 is also known to participate in transcriptional memory and in the regulation of immune response genes whose expression is stimulated by viral infections.^19-21^ In Drosophila cells, Nup98 has been shown to promote RNA Pol II occupancy at target gene promoters, poising them for a coordinated and rapid induction of the antiviral response.^19^ Several of these antiviral response genes are regulated by the transcription factor FoxK, which requires the presence of Nup98 for transcriptional induction.^20^ Additionally, human Nup98 plays a role in transcriptional memory of interferon-induced genes by interacting with their promoters. Specifically, interferon-induced gene promoters containing Nup98 accumulate poised RNA Pol II along with dimethylated histone H3K4, with depletion of Nup98 leading to the loss of this histone mark, an absence of poised RNA polymerase, and slowed re-induction of gene expression.^21^

Overall, these studies highlight various functions for FG-Nups in gene expression, focused here on Nup98, that are outside the canonical setting of NPCs and nucleocytoplasmic transport. We expect that FG-Nups perform these additional tasks by using the same domains and interaction surfaces functioning within NPCs. Consequently, we would argue that future work should focus on understanding the molecular details of these interactions, which are central to understanding how mutations in FG-Nups contribute to disease and are manipulated by viruses to support infection and replication.

## Nup98 binds DHX9 and regulates its RNA-dependent ATPase activity

The studies discussed above, and others, clearly establish the association of Nup98 with gene loci and a role for this protein in regulating their transcription. However, it remains to be determined what function(s) is performed by Nup98 in this context. Previous work has detected interactions between Nup98 and proteins with defined roles in regulating chromatin structure, such as the histone acetyltransferase CBP and the transcription factor FOXO1,^31,32^ but the consequences of these interactions remain to be defined. To further understand the possible mechanisms by which Nup98 might participate in gene expression, we focused on identifying intranuclear proteins that interact with Nup98. Using an immunoprecipitation and mass spectrometry approach, we identified candidate Nup98 interactors that, in addition to previously recognized binding partners (i.e. Nup88,^25^ Rae1,^33^ NXF1,^34^ and XPO1^35^), included multiple DBPs (i.e., DDX17, DDX21, DDX3, DDX5 and DHX9).^7^ Among these, DHX9 stood out based on the number of unique peptides and the presence of other proteins in the Nup98 immunoprecipitation that are known interactors of DHX9.^7^

DHX9, also known as RNA helicase A (RHA), is a member of the DExH/D-box protein family that is capable of using ATP to bind and remodel double stranded (ds) RNA or DNA-containing complexes.^36,37^ DHX9 has known regulatory roles in DNA replication, transcription, translation, RNA processing and transport, microRNA processing, and maintenance of genomic stability.^38^ Like other members of the DBP family, DHX9 contains 2 conserved RecA-like domains responsible for forming the helicase core.^38^ Flanking this region on the N-terminal side are 2 double-stranded RNA-binding domains (dsRBDs) and the minimal transactivation domain (MTAD), the site of RNA Pol II interaction.^38^ On the C-terminal side are an oligonucleotide/oligosaccharide-binding fold (OB-fold), nuclear localization and nuclear export sequences, and a glycine-rich RGG-box that can interact with single-stranded nucleic acids.^38^ The 2 dsRBDs have been shown to bind dsRNA and both the dsRBDs and RGG-box are required for the nucleic acid-stimulated ATPase activity of DHX9.^39^

**Figure 1.**
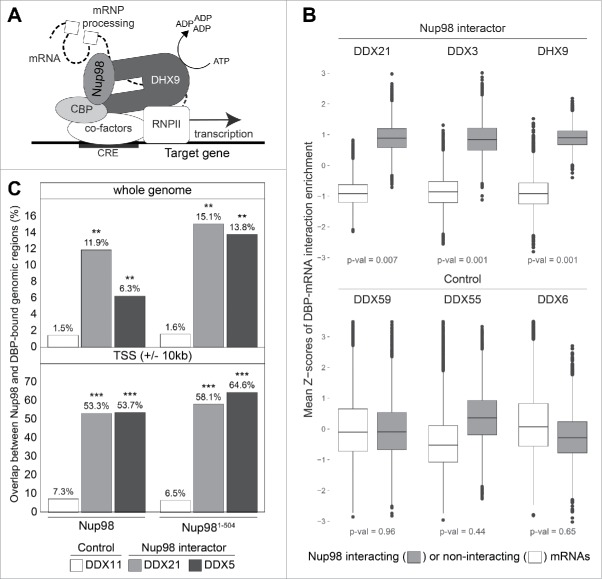
Nup98 and select DBPs interact with a shared set of mRNAs and gene loci. (A) Diagram depicting Nup98 (medium gray) and DHX9 (dark gray) at the promoter of a gene containing a cAMP responsive element (CRE) (indicated as a black box) participating in gene transcription and pre-mRNA processing. The diagram is based on the direct interactions of DHX9 with CBP (light gray), Nup98 with DHX9, and these factors with shared gene loci (black line) and transcripts (dashed line). In this model, Nup98 stimulates DHX9 ATPase activity to induce DHX9-mediated, CBP-dependent activation of RNA Pol II transcription, with Nup98-DHX9 further interacting with the pre-mRNA to regulate processing (e.g., splicing). (B) mRNA interaction data for select DBPs that bind (DDX21, DDX3, and DHX9)^54-56^ or do not bind (DDX59, DDX55 and DDX6)^59,60^ Nup98. Based on published RIP-seq data, the transcripts interacting with each DBP were categorized into Nup98-interacting (gray) or non-interacting (white) mRNAs.^53,61^ Bootstrapped means of mRNA enrichment for these 2 categories of DBP-interacting transcripts were calculated and transformed into Z-scores. Box plots show average Z-scores for mRNAs known to interact with Nup98 (gray) vs. those that show no Nup98 interaction (white), with the median represented by a horizontal line, 50% of the data present within the box, and the whiskers indicating the maximum and minimum values (outliers are shown as points). P-values were obtained by label permutation tests (Nup98 interacting or non-interacting mRNAs) via Monte-Carlo resampling and they are shown at the bottom of the graphs. (C) Analysis of published data^14,55,57,58^ to compare genomic regions bound to full length Nup98 or the nucleoplasmic Nup98^1-504^ truncation with DBPs detected (DDX21 and DDX5)^55,57^ or not detected (DDX11)^58^ with immunopurified Nup98.^7,61^ Plots show the percent overlap of genomic regions (peaks) in the entire genome (top) or at genomic regions close (+/− 10 kb) to the transcriptional start site (TSS) of genes (bottom) bound by full length Nup98 or Nup98^1-504^ compared with the indicated DBPs. Adjusted p-values indicating statistical significance of observed overlaps are shown as *** < 0.001 < ** < 0.01 and are indicated above each bar. For panels B and C, see ref.^61^ for further methodological information.

*In vivo*, several protein co-factors bind the auxiliary N- and C-terminal domains of DHX9 to regulate its ATPase activity, and, in turn, regulate the cellular functions of DHX9.^38,40^ Consistent with such a model of DHX9 regulation, we observed that Nup98 bound to both N- and C-terminal regions of DHX9 (residues 1-380, containing the dsRBDs and MTAD; and residues 821-1270, containing the OB-fold and RGG-box), through the FG repeat-containing region of Nup98. Most notably, the binding of Nup98 to DHX9 stimulated the ATPase activity of DHX9 in the presence of RNA, an observation that suggests Nup98 functions as a cofactor to regulate DHX9 activity (Fig. 1A).^7^

Other analysis of cofactors that regulate DBPs have identified domains with intriguing similarities to the FG-regions of Nup98. For example, the activity of the DBP Prp43 is stimulated through binding the G-patch domain of yeast Ntr1.^41,42^ Like the FG region of Nup98, a G-patch domain is intrinsically unstructured and rich in glycine and bulky hydrophobic (e.g., leucine) amino-acid residues.^41^ Interestingly, the Ntr1 G-patch has been proposed to adopt secondary structure features after binding Prp43.^42^ We envision that binding of Nup98 to DHX9 could similarly impart structure on the FG-region of Nup98 and produce a unique binding interface that, in turn, could regulate binding of the complex to specific chromatin loci and RNA species.^7^ This idea is consistent with our observations that the binding of certain mRNAs to Nup98 is facilitated by DHX9.^7^ Future structural analyses will be required to understand the consequences of Nup98-DHX9 complex formation on the structure and domain arrangements of both proteins. Such analyses may, more generally, provide insight into the function of plastic FG-domains in contexts outside of NPCs.

## Function of the Nup98-DHX9 complex

Both Nup98 and DHX9 have reported roles in regulating gene expression (reviewed in refs.^2,8,38,43^) and our analysis of associated chromatin and mRNAs showed that Nup98 and DHX9 physically associate with a shared set of gene loci and their transcribed mRNAs.^7^ Moreover, depletion of either protein reduced the interaction of the other protein to these gene loci and altered the expression patterns of these genes, which together suggests that Nup98 regulates DHX9 *in vivo*.^7^ Analysis of genes whose expression is altered upon depletion of Nup98 or DHX9 further revealed that >60% of these genes contained a putative cAMP-response element (CRE) (p = 1.16 × 10^−21^), and CRE-containing genes represent ∼50% of the Nup98 interacting gene loci detected in Nup98-Dam-ID studies (p = 5.5 × 10^−64^).^7,14,44,45^ Consistent with these observations, Nup98 has been reported to interact with the CREB-binding protein CBP,^31^ a transcriptional co-activator that is recruited to CRE-containing genes. DHX9 has also been shown to directly interact with CBP to aid in recruitment of RNA polymerase II to CRE-containing genes,^46^ with the ATPase activity of DHX9 being important for transcriptional induction of genes regulated by a CRE sequence.^47^ These data prompted us to test if Nup98 could stimulate CBP-dependent transcription in a manner consistent with its ability to stimulate the ATPase activity of DHX9, which we found was the case.^7^ These findings support a model in which Nup98 functions as a cofactor to regulate the transcriptional functions of DHX9 at a subset of genes (Fig. 1A).

In addition to being present at shared gene loci, Nup98 and DHX9 bind mRNA transcripts from these same genes,^7^ suggesting that a Nup98-DHX9 complex likely contributes to pre-mRNA processing, to which DHX9 has also previously been linked.^48,49^ Consistent with this, analysis of transcriptome data from cells depleted of DHX9 or Nup98 show 217 shared transcripts with splicing defects.^7^ Cells depleted of Nup98 or DHX9 also showed common splicing defects of the E1A reporter gene,^7^ which together suggests that DHX9 and Nup98 are involved in mRNA splicing. Moreover, analysis of several mRNAs that bind to Nup98 and DHX9 showed increased binding to DHX9 upon depletion of Nup98.^7^ We expect that this could result from a reduction in DHX9 ATPase activity and a resulting increase in DHX9 residence time on mRNA, since ATP hydrolysis likely precedes release of a bound RNA. This leads us to propose that DHX9 is loaded onto the transcribed pre-mRNA to facilitate mRNA splicing and the dynamics of this binding event are regulated by Nup98.^7^ We envisage that altering the dynamics of RNA-binding influences mRNA splicing by limiting DHX9 activity and/or interfering with downstream processing events. It is important to note that it is not currently known if the role of the Nup98-DHX9 complex in transcription and splicing are related or functionally independent.

## Nups and DBPs in gene expression regulation

The ability of Nup98 to bind and regulate the activity of DHX9 may reflect a broader role for Nup98, and possibly other FG-Nups, in regulating the activity of DBPs and modulating gene expression. For example, we detected several DBPs bound to Nup98 including DDX21, DDX5, DDX17, DHX15, and DDX3.^7^ Interestingly, Nup98 and this group of putative interacting DBPs have each been implicated in antiviral immune responses through sensing viral dsRNA and contributing to the expression of interferon and interferon-stimulated genes.^50-52^ Whether the binding of Nup98 to these DBPs regulates their function, as observed for DHX9, and plays a role in antiviral immunity remains to be investigated. However, an examination of published data sets^14,53-61^ revealed shared gene loci and mRNAs bound to Nup98 and those DBPs we identified in the Nup98 immunoprecipitation (DDX3, DDX5, DDX21; Fig. 1B and C). Furthermore, comparison of chromatin and mRNA interaction data for DDX21 and Nup98 indicates that 50% of mRNAs bound by Nup98 also interact with DDX21 (p-value 4.7 × 10^−234^), with 20% of these transcripts being encoded by genes that are also bound by both Nup98 and DDX21 (p-value 7.5 × 10^−26^). The repeating association of a DBP and Nup98 with shared genes and transcripts supports a potential role for Nup98 in modulating the activity of multiple DBPs and the diverse cellular functions that they facilitate. Notably, there are many other nucleoplasmic FG-Nups that have been shown to regulate gene expression (e.g., Nup50, Nup62, Nup153, sPom121),^14,16,28^ and determining whether these FG-Nups also bind and regulate DBPs promises to be an exciting area for future study.
